# Structure of TnsABCD transpososome reveals mechanisms of targeted DNA transposition

**DOI:** 10.1063/4.0000816

**Published:** 2025-10-27

**Authors:** Shukun Wang, Romana Siddique, Mark Hall, Phoebe Rice, Leifu Chang

**Affiliations:** 1 Purdue University, West Lafayette, Indiana, USA; 2 The University of Chicago, Chicago, Illinois, USA

## Abstract

Tn7-like transposons are characterized by their ability to insert specifically into host chromosomes. Recognition of the attachment (*att*) site by TnsD recruits the TnsABC proteins to form the transpososome and facilitate transposition. Although this pathway is well established, atomic-level structural insights of this process remain largely elusive. Here, we present the cryo- electron microscopy (cryo-EM) structures of the TnsC-TnsD- *att* DNA complex and the TnsABCD transpososome from the Tn7-like transposon in *Peltigera membranacea cyanobiont* 210A, a type I-B CRISPR-associated transposon. Our structures reveal a striking bending of the *att* DNA, featured by the intercalation of an arginine side chain of TnsD into a CC/GG dinucleotide step. The TnsABCD transpososome structure reveals TnsA-TnsB interactions and demonstrates that TnsC not only recruits TnsAB but also directly participates in the transpososome assembly. These findings provide mechanistic insights into targeted DNA insertion by Tn7- like transposons, with implications for improving the precision and efficiency of their genome-editing applications.

